# 67 Transient Dysphagia After Burn Injury in Children: An Under-identified Problem

**DOI:** 10.1093/jbcr/irac012.070

**Published:** 2022-03-23

**Authors:** Trang Bui, Ingrid Parry, Pauline W Ng, Kathleen S Romanowski, Tina L Palmieri, David Greenhalgh, Soman Sen

**Affiliations:** Shriners Hospitals for Children Northern California, Sacramento, California; Shriners Hospitals for Children Northern California, Sacramento, California; Shriners Hospital for Children - Northern CA, Sacramento, California; University of California, Davis and Shriners Hospitals for California Northern California, Sacramento, California; UC Davis and Shriners Hospitals for Children Northern California, Sacramento, California; ; UC Davis, Sacramento, California

## Abstract

**Introduction:**

Severely burn injured pediatric patients are at risk of dysphagia (difficulty swallowing) due to prolonged intubation or tracheostomy placement. To improve the early identification and treatment of dysphagia, we implemented a swallowing assessment protocol. We hypothesized that the swallowing assessment protocol is effective for identifying and treating dysphagia after prolonged intubation.

**Methods:**

Between October 2016 and December 2020, pediatric burn patients with facial burn injuries, prolonged mechanical ventilation, tracheostomy, inhalation injury and/or anoxic events were placed on the swallowing protocol. The protocol included a Transitional Swallow Screen (TSS) performed within 24 hours after extubation or decanulation by an advanced practice swallow occupational therapist. If signs of dysphagia were noted, recommendations on diet consistency and treatment for positioning and feeding were implemented. Regular reassessments continued until the patient was determined to have regained premorbid swallowing function. Data on patient demographics, burn characteristics, dysphagia, treatment and outcome were collected. Descriptive statistics were used to describe the population, treatments and outcome.

**Results:**

A total of 33 pediatric burn patients were included. Mean age was 8.1±5.9 years and TBSA was 48.1±26.8%. Median time from injury to swallow assessment was 45 (21-81) days. The majority of patients suffered from flame burns (70%). Almost all of the patients were intubated (97%) and 85% underwent a tracheostomy. Patients had a facial burn (73%), inhalation injury (24%) or anoxic injury (15%). Transient dysphagia was diagnosed in 79% of patients. Subsequent therapeutic procedures as a result of the TSS included: neurologic re-education (30%), swallow therapy exercises (55%), desensitization (42%), and patient/ family training and supervision (79%). All patients eventually returned to normal swallow and regular diet. This occurred at an average of 72.5+46.7 days post injury and 8.2+18.0 days post swallow assessment.

**Conclusions:**

Pediatric patients with substantial burn injury may not only be at risk for aspiration but also have other forms of dysphasia that require intervention. Implementation of a swallowing protocol can identify patients who required further therapeutic intervention and can guide the recovery of safe swallowing and functional oral intake.

